# Functional and Comparative Genomic Analysis of Integrated Prophage-Like Sequences in “*Candidatus* Liberibacter asiaticus”

**DOI:** 10.1128/mSphere.00409-19

**Published:** 2019-11-13

**Authors:** Marian Dominguez-Mirazo, Rong Jin, Joshua S. Weitz

**Affiliations:** a School of Biological Sciences, Georgia Institute of Technology, Atlanta, Georgia, USA; b Interdisciplinary Graduate Program in Quantitative Biosciences, Georgia Institute of Technology, Atlanta, Georgia, USA; c School of Physics, Georgia Institute of Technology, Atlanta, Georgia, USA; University of Wisconsin—Madison

**Keywords:** One Health, bioinformatics, environmental microbiology, microbial ecology, phage ecology, phytopathology, plant pathogens

## Abstract

Huanglongbing disease (HLB; yellow shoot disease) is a severe worldwide infectious disease for citrus family plants. The pathogen “*Candidatus* Liberibacter asiaticus” is an alphaproteobacterium of the *Rhizobiaceae* family that has been identified as the causative agent of HLB. The virulence of “*Ca.* Liberibacter asiaticus” has been attributed, in part, to prophage-carried genes. Prophage and prophage-like elements have been identified in 12 of the 15 available “*Ca.* Liberibacter asiaticus” genomes and are classified into three prophage types. Here, we reexamined all 15 “*Ca.* Liberibacter asiaticus” genomes using a *de novo* prediction approach and expanded the number of prophage-like elements from 16 to 33. Further, we found that all of the “*Ca.* Liberibacter asiaticus” genomes contained at least one prophage-like sequence. Comparative analysis revealed a prevalent, albeit previously unknown, prophage-like sequence type that is a remnant of an integrated prophage. Notably, this remnant prophage is found in the Ishi-1 “*Ca.* Liberibacter asiaticus” strain that had previously been reported as lacking prophages. Our findings provide both a resource for data and new insights into the evolutionary relationship between phage and “*Ca.* Liberibacter asiaticus” pathogenicity.

**IMPORTANCE** Huanglongbing (HLB) disease is threatening citrus production worldwide. The causative agent is “*Candidatus* Liberibacter asiaticus.” Prior work using mapping-based approaches identified prophage-like sequences in some “*Ca.* Liberibacter asiaticus” genomes but not all. Here, we utilized a *de novo* approach that expands the number of prophage-like elements found in “*Ca.* Liberibacter asiaticus” from 16 to 33 and identified at least one prophage-like sequence in all “*Ca.* Liberibacter asiaticus” strains. Furthermore, we identified a prophage-like sequence type that is a remnant of an integrated prophage—expanding the number of prophage types in “*Ca.* Liberibacter asiaticus” from 3 to 4. Overall, the findings will help researchers investigate the role of prophage in the ecology, evolution, and pathogenicity of “*Ca.* Liberibacter asiaticus.”

## Abstract (precis)

Huanglongbing (HLB) disease is threatening citrus production worldwide. The causative agent is “*Candidatus* Liberibacter asiaticus.” Prior work using mapping-based approaches identified prophage-like sequences in some “*Ca.* Liberibacter asiaticus” genomes but not all. Here, we utilized a *de novo* approach that expands the number of prophage-like elements found in “*Ca.* Liberibacter asiaticus” from 16 to 33 and identified at least one prophage-like sequence in all “*Ca.* Liberibacter asiaticus” strains. Furthermore, we identified a prophage-like sequence type that is a remnant of an integrated prophage—expanding the number of prophage types in “*Ca.* Liberibacter asiaticus” from 3 to 4. Overall, the findings will help researchers investigate the role of prophage in the ecology, evolution, and pathogenicity of “*Ca.* Liberibacter asiaticus.”

## INTRODUCTION

“*Candidatus* Liberibacter asiaticus” has been identified as one of the three “*Ca.* Liberibacter” species that cause Huanglongbing disease (HLB; yellow shoot disease [also known as citrus greening disease]). HLB is a major threat to the worldwide citrus-growing industry (1). Symptoms of infected trees include leaf mottling, deformed/discolored fruits, premature fruit drop, and premature mortality (2). It has been suggested that “*Ca.* Liberibacter asiaticus”-encoded proteins have an inhibitory effect in plant defenses (3, 4), but comprehensive understanding of the mechanism of infection is still lacking. No effective disease management practice is currently available. Hence, understanding the genomic composition of “*Ca.* Liberibacter asiaticus” and its infection mechanisms is likely to help develop strategies to manage the disease.

As “*Ca.* Liberibacter asiaticus” has not been cultivated *in vitro*, its biological study has been based on analyses of DNA extracted from infected plants or insects. To date, a total of 15 genome assemblies have been made available at the National Center for Biotechnology Information (NCBI) Genome database (Table 1). The “*Ca.* Liberibacter asiaticus” genome size is ∼1.2 Mb, with G+C content of 36.5%; both the small genome and low level of G+C content are consistent with patterns of obligate intracellular bacteria (5). The “*Ca.* Liberibacter asiaticus” genome includes genes involved in cell motility and active transport (5, 6); despite the presence of these genes, only passive movement has been observed in the phloem sap (6). Furthermore, prophage sequences have also been identified in multiple “*Ca.* Liberibacter asiaticus” strains (7–16) (Tables 1 and 2). The interest in “*Ca.* Liberibacter asiaticus” prophage stems from observations revealing that a prophage-encoded peroxidase is an effector that suppresses plant defenses (3). However, strains reported to lack prophages still induce HLB symptoms (8, 16). Hence, it has been hypothesized that prophages might contribute to bacterial virulence but are not required for “*Ca.* Liberibacter asiaticus” pathogenicity (6).

**TABLE 1:** “*Ca.* Liberibacter asiaticus” genome information^*a*^

Strain	Source	GenBank accession no.	No. of contigs	Length (bp)	G+C (%)	Prophage type(s)
A4	Guangdong, China	GCA_000590865.2	—	1,233,514	36.4	2
AHCA1	California, USA	GCA_003143875.1	—	1,233,755	36.63	1
FL17	Florida, USA	GCA_000820625.1	3	1,227,253	36.47	1
gxpsy	Guangxi, China	GCA_000346595.1	—	1,268,237	36.57	1, 2
HHCA1	California, USA	GCA_000724755.2	239	1,150,620	36.55	2*
Ishi-1	Okinawa, Japan	GCA_000829355.1	—	1,190,853	36.32	
JXGC	Jiangxi, China	GCA_002216815.1	—	1,225,162	36.4	3*
psy62	Florida, USA	GCA_000023765.2	—	1,227,328	36.47	1, 2*
SGCA1	California, USA	GCA_003149415.1	606	233,414	36.25	1
SGCA5	California, USA	GCA_001430705.1	56	1,201,385	36.36	1*
SGpsy	California, USA	GCA_003336865.1	1,402	769,888	36.32	1
TX1712	Texas, USA	GCA_003160765.1	48	1,203,333	36.36	
TX2351	Texas, USA	GCA_001969535.1	71	1,252,002	36.52	
YCPsy	Guangdong, China	GCA_001296945.1	9	1,233,647	36.48	1, 3
YNJS7C	Yunnan, China	GCA_003615235.1	3	1,258,986	36.58	2, 3

a Characteristics of the 15 publicly available “*Ca.* Liberibacter asiaticus” genomes are indicated as follows: strain, geographic origin, GenBank accession number, number of contigs, sequence length, G+C content, and associated phage types. Dashes (—) in the contig column indicate complete (circularized) genomes. Phage types with asterisks belong to phages with publicly available genomes (Table 2).

**TABLE 2:** “*Ca.* Liberibacter asiaticus” phage genome information^*a*^

Phage	Strain	GenBank accession no.	No. of contigs	Length (bp)	G+C (%)	Type
SC1	UF506	GCA_000901995.1	—	40,048	41.2	1
SC2	UF506	GCA_000903515.1	—	38,997	39.38	2
P-JXGC-3	JXGC	GCA_002620145.1	—	31,449	39.97	3
P-HHCA1-2	HHCA1	GCA_002614125.1	—	38,989	39.31	2
FP2	psy62	GCA_002630885.1	—	38,552	39.27	2
P-SGCA5-1	SGCA5	GCA_002945115.1	2	37,487	41.85	1

a Characteristics of the 5 publicly available genomes of “*Ca.* Liberibacter asiaticus” phages are indicated as follows: phage name, associated “*Ca.* Liberibacter asiaticus” strain, GenBank accession number, number of contigs, sequence length, G+C content, and phage type. Dashes (—) in the contig column correspond to closed genomes.

Prophage regions in “*Ca.* Liberibacter asiaticus” genomes are highly variable relative to the rest of the genome (8, 16). Comparative analyses of prophage sequences suggest endemism in “*Ca.* Liberibacter asiaticus” strains (14, 17). Based on currently available sequence data, “*Ca.* Liberibacter asiaticus” prophages have been classified as uniquely belonging to one of three types, i.e., type 1, 2, or 3. The first reported “*Ca.* Liberibacter asiaticus” prophages were SC1 and SC2 (type 1 and 2 prophages, respectively). They were discovered when the sequencing of a “*Ca.* Liberibacter asiaticus” strain showed circular contigs rich in phage open reading frames (ORFs) (7). These phages were shown to integrate into the “*Ca.* Liberibacter asiaticus” genome (7). Moreover, a recent study identified P-JXGC-3 as the first type 3 prophage by using BLAST searches with phages SC1 and SC2 as query to locate phage-containing contigs within a “*Ca.* Liberibacter asiaticus” genome with incomplete read mapping to type 1 or type 2 prophage (13). The genomes of prophages of types 1, 2, and 3 can be divided into an early gene region and a late gene region (7, 13). The early gene regions of the three prophage types are highly similar and match about 50% of their genomes. In contrast, the late regions differ greatly in gene content between types (7, 13).

Current methods to identify prophage in “*Ca.* Liberibacter asiaticus” genomes rely on read mapping against known prophages (9, 12). As a consequence, failure of read mapping has been interpreted to mean that a genome is prophage free (e.g., as in the Ishi-1 strain [16]). More generally, we are unaware of any systematic analysis of prophage content in “*Ca.* Liberibacter asiaticus” genomes using *de novo* prediction approaches. In this study, we examined the genome sequences of 15 “*Ca.* Liberibacter asiaticus” strains and extracted phage sequences using *de novo* prediction tools, including Virsorter (18) and PHASTER (19). *De novo* prediction tools identify putative phage and prophage regions through detection of circular sequences and comparison of predicted proteins against complete phage databases (18, 19). In contrast to commonly used read-mapping methodologies, a *de novo* approach can identify phage regions that do not necessarily resemble “*Ca.* Liberibacter asiaticus” prophages of type 1, 2, or 3. Via a *de novo* approach, we identified several potential prophage elements not available in databases, of which 5 belonged to type 1, 2, or 3 “*Ca.* Liberibacter asiaticus” phage elements that had not been previously reported. We also identified 12 phage-like sequences present in all strains that do not resemble any previously identified phage type. Subsequent analyses revealed that these 12 sequences do not match type 1, 2, or 3 prophages. We argue that, based on composition and evolutionary analysis, it is likely that these sequences belong to a different “*Ca.* Liberibacter asiaticus” prophage-like sequence, which we term “type 4.” Multiple lines of evidence suggest that type 4 prophage-like sequences are remnants of an integrated prophage. This study expanded the number of prophage-like elements from 16 to 33. The results will provide a genomic resource for future investigations into the role of prophage in shaping the ecology, evolution, and pathogenicity of “*Ca.* Liberibacter asiaticus.”

## RESULTS

### Identification of novel prophage-like sequences.

A total of 35 putative prophage-like sequences were identified among the 15 “*Ca.* Liberibacter asiaticus” genomes by Virsorter and PHASTER. At least one sequence was predicted for each of the strains studied (Table 3) except for the genomes SGCA1 and SGpsy, where the absence of such a result might have been due to the high level of fragmentation of their assemblies (Table 1). Predicted sequences differ greatly in length, with the shortest sequence having ∼2,000 bp and the longest over 65,000 bp. The prophage-like sequences can be divided into two groups differing by the level of G+C content: those with around 40% (similar to the G+C content of type 1, 2, and 3 prophages) and those with around 36% (similar to the “*Ca.* Liberibacter asiaticus” value). We compared the predicted sequences to phage genomes available from NCBI (Table 2). Of the 35 predicted sequences, 6 belong to reported phage genomes—corresponding to 3 prophage sequences of HHCA1, 1 of JXGC, 1 of psy62, and 1 of SGCA5, belonging to phages P-HHCA1-2, P-JXGC-3, FP2, and P-SGCA5-1, respectively. Hence, we identified 29 prophage-like sequences that are not part of a publicly available prophage genome.

**TABLE 3:** Putative characteristics of prophage sequences^*a*^

Strain	Sequence ID	Contig	Position	Length (bp)	G+C content (%)
A4	A4-a	—	1130506–1149463	18,958	35.31
A4	A4-b	—	1189717–1232852	43,136	39.52
AHCA1	AHCA1-a	—	1135796–1196595	60,800	36.66
FL17	FL17-a	1	1132100–1197885	65,786	36.54
FL17	FL17-b	2	109–15184	15,076	43.39
FL17	FL17-c	3	76–13493	13,418	39.75
gxpsy	gxpsy-a	—	1118915–1137878	18,964	35.36
gxpsy	gxpsy-b	—	1178134–1226132	47,999	39.28
gxpsy	gxpsy-c	—	1226133–1266293	40,161	41.5
HHCA1	HHCA1-a	217	189–6282	6,094	37.12
HHCA1	HHCA1-b	229	160–8846	8,687	41.56
HHCA1	HHCA1-c	231	11–13045	13,035	40.84
HHCA1	HHCA1-d	239	11351–39707	28,357	39.26
Ishi-1	Ishi-1-a	—	1127556–1144572	17,017	35.31
JXGC	JXGC-a	—	1130487–1149448	18,962	35.35
JXGC	JXGC-b	—	1189703–1224971	35,269	40.2
psy62	psy62-a	—	948–11549	10,602	40.4
psy62	psy62-b	—	1180444–1226282	45,839	40.1
psy62	psy62-c	—	1132029–1149043	17,015	35.3
SGCA5	SGCA5-a	22	802–4833	4,032	37.03
SGCA5	SGCA5-b	43	3028–17448	14,421	44.39
TX1712	TX1712-a	3	51093–68108	17,016	35.3
TX1712	TX1712-b	17	127–27513	27,387	41.8
TX1712	TX1712-c	35	1026–7842	6,817	41.94
TX2351	TX2351-a	66	677–10598	9,922	41.14
TX2351	TX2351-b	67	438–11367	10,930	39.11
TX2351	TX2351-c	69	1515–7899	6,385	37.79
TX2351	TX2351-d	70	288–13018	12,731	40.6
YCPsy	YCPsy-a	2	692231–711192	18,962	35.35
YCPsy	YCPsy-b	3	13–21283	21,271	43
YCPsy	YCPsy-c	6	30–2207	2,178	39.99
YCPsy	YCPsy-d	7	1952–7990	6,039	41.91
YNJS7C	YNJS7C-a	1	1125779–1142794	17,016	35.31
YNJS7C	YNJS7C-b	2	735–39556	38,822	41.79
YNJS7C	YNJS7C-c	3	12161–31475	19,315	39.81

a Characteristics of the sequences predicted by Virsorter and PHASTER are indicated as follows: “*Ca.* Liberibacter asiaticus” strain, sequence ID, the contig in which the sequence was found, the position in contig, the sequence length, and percent G+C content. Dashes (—) in the contig column indicate that the corresponding genome is fully assembled.

### Classification of prophage-like predicted sequences.

Of the 29 predicted prophage-like sequences, a total of 13 were successfully classified into type 1, 2, or 3 (see Materials and Methods and Table 4). As previously described, strains psy62, gxpsy, FL17, and YCPsy have sequences classified as type 1. Strains A4 and gxpsy have sequences classified as type 2. Strain YNJS7C harbors a sequence that resembles a type 3 prophage (12, 15, 20). Novel prophage-like sequences of type 1 were found in strains TX1712 and TX2351, while type 2 sequences were also found in strain TX2351. In contrast to a previous report, no YNJS7C sequences were classified as type 2 (15); instead, a type 1 sequence was identified (see Addendum in Proof below). Although they seemed to resemble the representative phages, 4 sequences remained unclassified due to their small size. Finally, 12 sequences shared similar characteristics, but 10 had no apparent resemblance to the representative phages SC1, SC2, and P-JXGC-3, while a small subsequence of each of the other two (AHCA1-a and FL17-a) partially resembled the representative phages (Table 4).

**TABLE 4:**
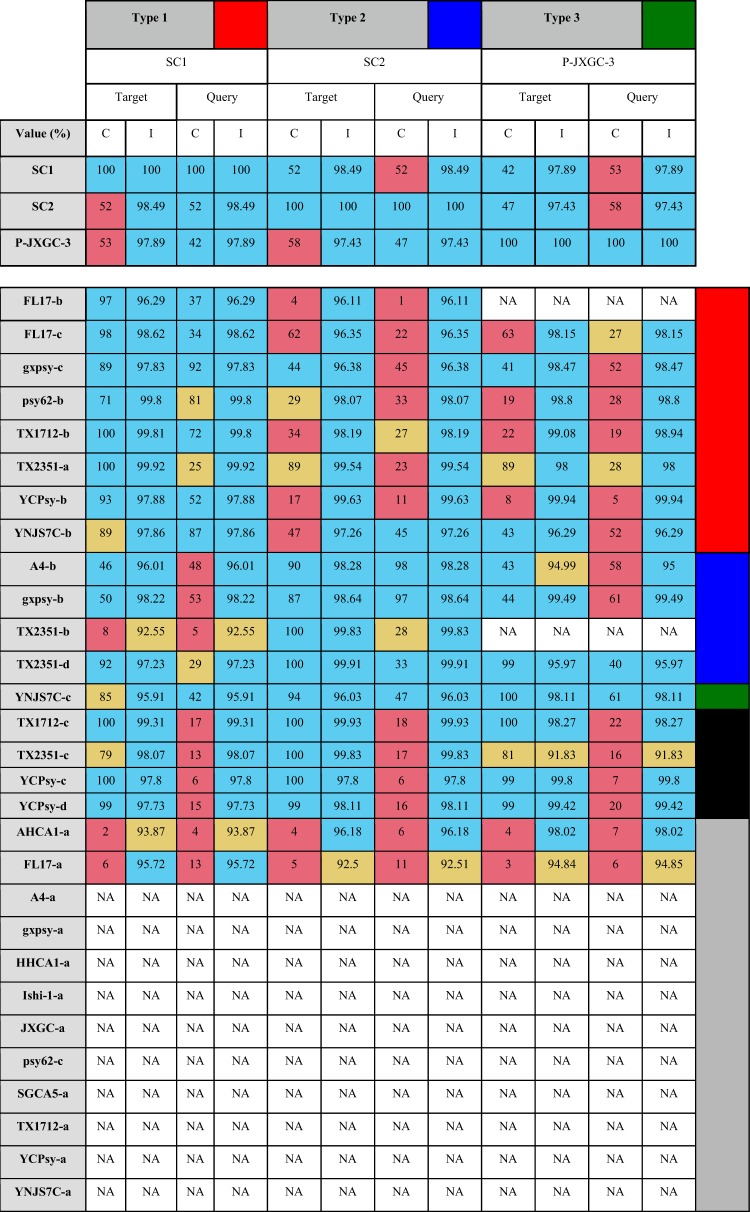
Classification of novel prophage-like sequences into types 1, 2, and 3^*a*^

a The novel predicted sequences were aligned against representative phages SC1, SC2, and P-JXGC-3 of types 1, 2, and 3. Coverage (C) values corresponding to two-way local alignments are presented in this table. Blue, mustard, and pink cells stand for high, intermediate, and low alignment values. Note that the threshold values used to classify coverage (C) vary according to sequence length and position in BLAST (either query or target) (see Materials and Methods). White cells with “NA” entries represent absence of significant hits. Sequences were categorized as type 1 (red), type 2 (blue), or type 3 (green) sequences; unclassified sequences with resemblance to the representative phages (black); or unclassified without resemblance (gray).

### Identification of a new type of “*Ca.* Liberibacter asiaticus” prophage-like sequences.

We next set out to identify the “type” of prophage for the unclassified prophage-like sequences found via our *de novo* approach. Of the 12 unclassified sequences that do not resemble the three known phage types, 10 are highly similar to each other and belong to a candidate type 4 prophage-like sequence that includes the Ishi-1 prophage prediction (Table 5). Our evidence that these prophage-like sequences belong to a new type is as follows. First, there is no apparent resemblance to prophages of type 1, 2, or 3 at the nucleotide level or at the amino acid level. Second, the length of the type 4 sequences is about half the size of representative phages of types 1, 2, and 3 (Table 6). Third, in contrast to the previously known prophage types (with G+C content of ∼40%), type 4 sequences have G+C content of 35% to 37%, which is closer to that of the host, whose G+C content is ∼36% (Tables 1 and 6).

**TABLE 5:**
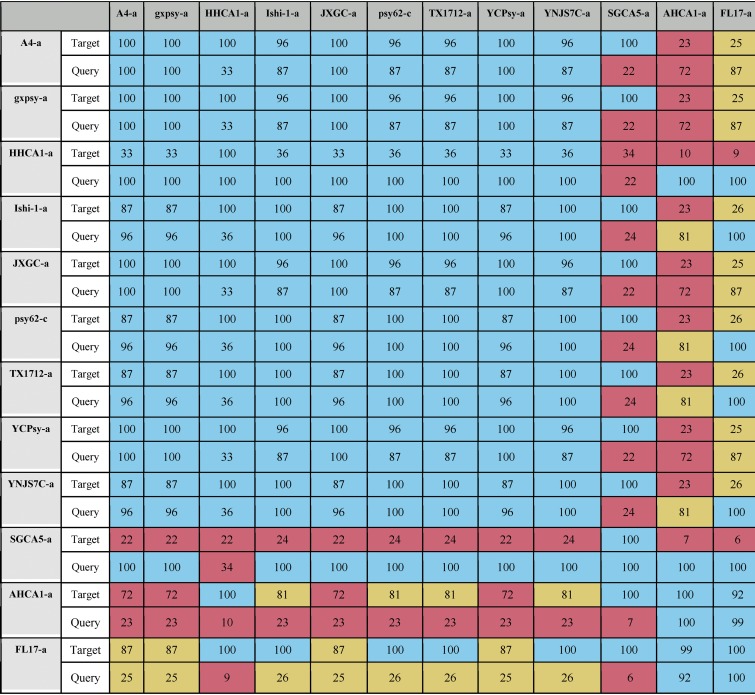
Resemblance between new type 4 sequences^*a*^

a Data represent results of comparisons of 12 predicted sequences that did not resemble any of the representative phages of types 1, 2, and 3. Coverage (C) values corresponding to two-way local alignments are presented in this table. Blue, mustard, and pink cells stand for high, intermediate, and low values, respectively (see Materials and Methods).

**TABLE 6:** Putative characteristics and classification of prophage sequences^*a*^

Sequence	Length (bp)	G+C content (%)	Associated name	Prophage type
FL17-a/F2*	4,021	37.8	P-FL17-1	1
FL17-b	15,076	43.39	P-FL17-1	1
FL17-c	13,418	39.75	P-FL171-1	1
gxpsy-c	40,161	41.5	P-gxpsy-1	1
psy62-b	45,839	40.1	FP1 / P-psy62-1	1
SGCA5-b	14,421	44.39	P-SGCA5-1	1
TX1712-b	27,387	41.8	P-TX1712-1	1
TX2351-a	9,922	41.14	P-TX2351-1	1
YCPsy-b	21,271	43	P-YCPsy-1	1
YNJS7C-b	38,822	41.79	P-YNJS7C-1	1
A4-b	43,136	39.52	P-A4-2	2
gxpsy-b	47,999	39.28	P-gxpy-2	2
HHCA1-b	8,687	41.56	P-HHCA1-2	2
HHCA1-c	13,035	40.84	P-HHCA1-2	2
HHCA1-d	28,357	39.26	P-HHCA1-2	2
psy62-a	10,602	40.4	FP2 / P-psy62-2	2
TX2351-b	10,930	39.11	P-TX2351-2	2
TX2351-d	12,731	40.6	P-TX2351-2	2
AHCA1-a/F2*	2,334	39.3	P-AHCA1-3	3
JXGC-b	35,269	40.2	P-JXGC-3	3
YNJS7C-c	19,315	39.81	P-YNJS7C-3	3
A4-a	18,958	35.31		4
gxpsy-a	18,964	35.36		4
HHCA1-a	6,094	37.12		4
Ishi-1-a	17,017	35.31		4
JXGC-a	18,962	35.35		4
psy62-c	17,015	35.3		4
TX1712-a	17,016	35.3		4
YCPsy-a	18,962	35.35		4
YNJS7C-a	17,016	35.31		4
AHCA1-a/F1*	13,713	35.5		4
FL17-a/F1*	14,507	35.4		4
SGCA5-a	4,032	37.03		4
TX1712-c	6,817	41.94		Unknown
TX2351-c	6,385	37.79		Unknown
YCPsy-c	2,178	39.99		Unknown
YCPsy-d	6,039	41.91		Unknown

a Characteristics of the predicted sequences are indicated as follows: sequence ID, length, G+C content, name (either previously assigned or assigned following the scheme proposed in reference 12), and prophage or prophage-like sequence type. Asterisks (*) mark sequences AHCA1-a/F1 and AHCA1-a/F2, which represent different fragments of sequence AHCA1-a, or sequences FL17-a/F1 and FL17-a/F2, which represent different fragments of sequence FL17-a.

**FIG 1:**
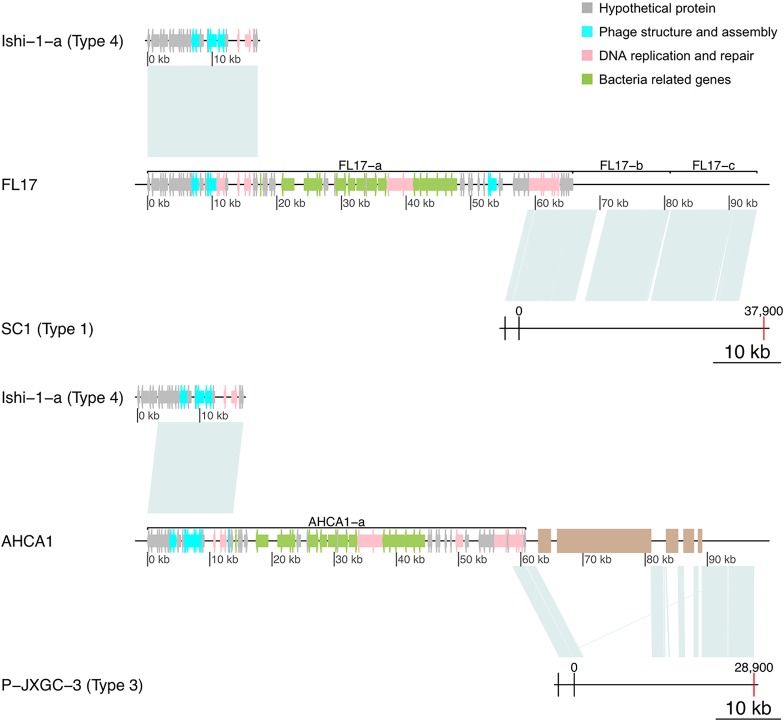
Reconstruction of type 1 and 3 prophage-like sequences using AHCA1-a and FL17-a and nearby regions. (Top panel) Sequence FL17-a is formed by a type 4 sequence followed by bacterial genes and a region resembling a type 1 prophage. Joining sequences FL17-a, FL17-b, and FL17-c reconstructs a type 1 prophage sequence with the majority of its organization similar to that predicted for the integration of SC1 (7). (Bottom panel) AHCA-1 contains a type 4 sequence followed by bacterial genes and a region highly resembling a type 3 prophage. Using nearby regions in the AHCA1 genome, a type 3 prophage can be reconstructed with an organization similar to that predicted for the integration of P-JXGC-3 (13). Brown rectangles represent assembly gaps. Alignment visualizations were done using the R package genoPlotR (v0.8.9).

The other 2 predicted sequences, FL17-a and AHCA1-a, contain ∼80% of the type 4 sequence (Table 5) followed by bacterial genes and fragments highly resembling a part of a type 1 prophage genome and a part of a type 3 prophage genome, respectively (Fig. 1 and Table 4). Table S1 in the supplemental material shows alignment values for the 12 unclassified sequences using the type 4 region of AHCA1-a and FL17-a.

10.1128/mSphere.00409-19.1 TABLE S1 Resemblance of new type 4 sequences. Results of cross-comparisons of the 12 predicted sequences that did not resemble any of the representative phages of types 1, 2, and 3 are presented. Putative prophage sequences AHCA1-a and FL17-a were cleaved, and the fragment from each resembling the other sequences was used. Identity (I) and coverage (C) values of two-way local alignments are presented on this table. Blue, mustard, and pink cells stand for high, intermediate, and low values, respectively (see Materials and Methods). All sequences were classified as new prophage-like sequence type 4. Download Table S1, DOCX file, 0.2 MB. Copyright © 2019 Dominguez-Mirazo et al. 2019 Dominguez-Mirazo et al. This content is distributed under the terms of the Creative Commons Attribution 4.0 International license.

The prophage fragment at the end of FL17-a (end of contig 1) joined to type 1 sequences FL17-b (contig 2) and FL17-c (contig 3) reconstructs the sequence of a type 1 prophage (Fig. 1, top panel) with a sequence organization similar to that reported for the chromosomal integration of phage SC1 (7). Likewise, the last part of the AHCA1-a sequence and the following region found after a genome assembly gap reconstruct a type 3 prophage with the sequence organization predicted for P-JXGC-3 (13) (Fig. 1, bottom panel). Only a type 1 prophage has been reported in strain AHCA1 (9), but the sequence organization of the integrated prophage (Fig. 1) and the presence of a hypothetical protein (GenBank accession no. YP_007011137.1) found only in prophages of type 2 and 3 (see Table S2) suggest that “*Ca.* Liberibacter asiaticus” strain AHCA1 harbors a type 3 prophage sequence. The truncation of the type 1 and type 3 prophage sequences and their closeness to the type 4 prophage-like sequence in strains FL17 and AHCA1 explain the incorrect concatenation of these sequences into a single prediction by Virsorter and PHASTER. Table 6 shows all predicted prophage-like sequences with their assigned type and sequence characteristics.

10.1128/mSphere.00409-19.2 TABLE S2 AHCA1-a gene function. ORFs predicted for AHCA1-a were annotated using BLASTp against the nonredundant database. Information includes gene start and end positions, UniRef ID, and predicted gene function. The corresponding locus_tag from the GenBank AHCA1 genome annotation is presented in the succeeding columns with the locus_tag ID, start and end positions in the bacterial genome, and annotated function. Note that some ORFs lack a corresponding locus_tag and that some locus_tags lack a corresponding ORF. Download Table S2, DOCX file, 0.2 MB. Copyright © 2019 Dominguez-Mirazo et al. 2019 Dominguez-Mirazo et al. This content is distributed under the terms of the Creative Commons Attribution 4.0 International license.

### Pan-genome analysis.

**FIG 2:**
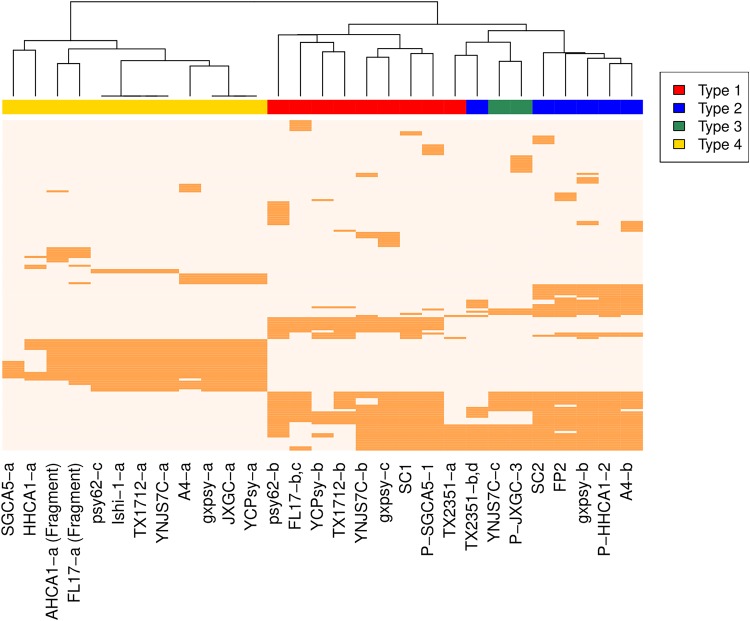
Gene composition in prophage-like sequences. A presence-absence heatmap of predicted pangenome proteins for the prophage-like sequences and phage available in public databases is shown. A clustering approach was used to obtain gene content relationships between sequences. Colors denote sequence classification as follows: red, type 1; blue, type 2; green, type 3; yellow, type 4. Sequences from the same strain that were classified as the same phage type, e.g., FL17 type 1 and TX2351 type 2, were used as a single phage for gene content analysis. Unclassified sequences were excluded from the analysis.

In order to evaluate the robustness of the classification identified in prior sections, we analyzed the pan-genome content of prophages through a clustering approach (Fig. 2). Clustering of gene content in prophage reveals a separation between the 4 types of sequences. All type 1 sequences cluster together except for TX2351-a. Type 2 and type 3 sequences appear nearby in the clustering. The type 2 putative prophage for strain TX2351 clusters with type 3 sequences. The two mistaken assignments might result from the short size of sequences and the overall close relationship between prophage types 1, 2, and 3. All sequences of type 4 cluster together as the most distant group. The clustering of prophage-like sequences based on gene content predicts relationships that roughly agree with the classification.

Table 7 presents a summary of the associated prophage elements in the 15 “*Ca.* Liberibacter asiaticus” strains, including those previously reported and the ones introduced in this study. There are 5 newly reported elements that belong to prophages of type 1, 2, or 3 (see “Prophage-like sequence identification” section below) and 12 type 4 prophage-like sequences. Our findings expand the number of known prophage elements in “*Ca.* Liberibacter asiaticus” from 16 to 33.

**TABLE 7:** “*Ca.* Liberibacter asiaticus” genome information adding new prophage-like sequences^*a*^

Strain	Source	GenBank accession no.	No. of contigs	Length (bp)	GC (%)	Prophage type(s)
A4	Guangdong, China	GCA_000590865.2	—	1,233,514	36.4	2, ***4***
AHCA1	California, USA	GCA_003143875.1	—	1,233,755	36.63	1, ***3***, ***4***
FL17	Florida, USA	GCA_000820625.1	3	1,227,253	36.47	1, ***4***
gxpsy	Guangxi, China	GCA_000346595.1	—	1,268,237	36.57	1, 2, ***4***
HHCA1	California, USA	GCA_000724755.2	239	1,150,620	36.55	2, ***4***
Ishi-1	Okinawa, Japan	GCA_000829355.1	—	1,190,853	36.32	***4***
JXGC	Jiangxi, China	GCA_002216815.1	—	1,225,162	36.4	3, ***4***
psy62	Florida, USA	GCA_000023765.2	—	1,227,328	36.47	1, 2, ***4***
SGCA1	California, USA	GCA_003149415.1	606	233,414	36.25	1
SGCA5	California, USA	GCA_001430705.1	56	1,201,385	36.36	1, ***4***
SGpsy	California, USA	GCA_003336865.1	1,402	769,888	36.32	1
TX1712	Texas, USA	GCA_003160765.1	48	1,203,333	36.36	***1***, ***4***
TX2351	Texas, USA	GCA_001969535.1	71	1,252,002	36.52	***1***, ***2***
YCPsy	Guangdong, China	GCA_001296945.1	9	1,233,647	36.48	1, 3, ***4***
YNJS7C	Yunnan, China	GCA_003615235.1	3	1,258,986	36.58	***1***, 2, 3, ***4***

a Characteristics of the 15 publicly available “*Ca.* Liberibacter asiaticus” genomes are indicated as follows: strain, geographic origin, GenBank accession number, number of contigs, sequence length, G+C content, and associated prophage types. Prophage types identified in this study are indicated in bold and italics. Dashes (—) in the number of contigs column indicate complete (circularized) genomes.

### Functional annotation of type 4 prophage-like sequences.

**FIG 3:**
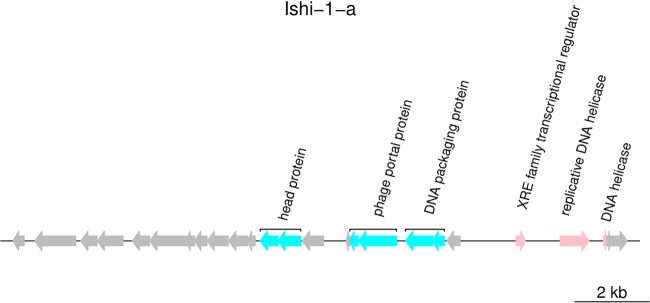
Annotation of representative sequence Ishi-1-a of type 4. The figure represents predicted CDS of Ishi-1-a, with ORFs color coded to predicted functions.

In order to identify characteristic features of type 4 “*Ca.* Liberibacter asiaticus” prophage-like sequences, we functionally annotated the representative prophage-like sequence Ishi-1-a (Fig. 3). Annotation of Ishi-1-a revealed 26 putative coding DNA sequences (CDS). Of them, 14 ORFs corresponded to hypothetical proteins. Moreover, Ishi-1-a contains several ORFs that present premature stop codons. These ORFs code mainly for fragments of genes with phage structure and assembly functions. Table S3 contains the detailed list of the Ishi-1-a sequence annotation.

10.1128/mSphere.00409-19.3 TABLE S3 Ishi-1-a gene function. ORFs predicted for Ishi-1-a were annotated using BLASTp against the nonredundant database. Information includes gene start and end positions in Ishi-1-a, UniRef ID, and predicted gene function. The corresponding locus_tag from the GenBank Ishi-1-a genome annotation is presented in the succeeding columns with the locus_tag ID, start and end positions in the bacterial genome, and annotated function. Note that some ORFs lack a corresponding locus_tag and that some locus_tags lack a corresponding ORF. Download Table S3, DOCX file, 0.2 MB. Copyright © 2019 Dominguez-Mirazo et al. 2019 Dominguez-Mirazo et al. This content is distributed under the terms of the Creative Commons Attribution 4.0 International license.

In Ishi-1, the sequence of a head protein found in “*Candidatus* Liberibacter solanacearum” is fragmented into ORF12 and ORF13. Three CDS (ORF15, ORF16, and ORF17) resemble different parts of a portal protein found also in “*Ca.* Liberibacter solanacearum.” ORF18 and ORF19 are remnants of a DNA packaging protein similar to one present in “*Candidatus* Liberibacter africanus.” There are three ORFs involved in DNA replication: a gene that codes for a transcriptional regulator of the XRE family (ORF22) and two fragments (ORF23 and ORF24) of a helicase coding gene. Furthermore, no attachment sites were found in the bacterial genome near the type 4 sequences.

The disruption of viral-particle-forming genes, along with the absence of att sites, the G+C content closely resembling that of the bacterial host, and the small sequence size, implies that type 4 sequences are remnants of a prophage integrated into the bacterial genome.

### Evolutionary origins of prophage-like sequence type 4 in “*Ca.* Liberibacter asiaticus.”

**FIG 4:**
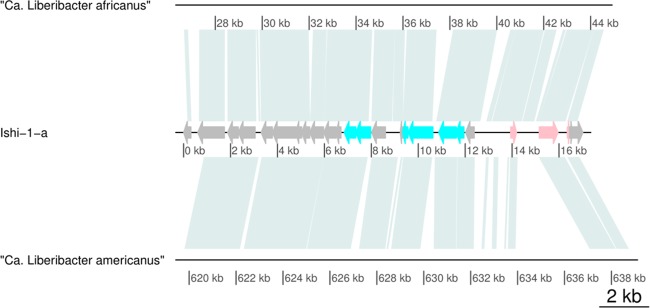
Presence of prophage-like sequence type 4 in related “*Candidatus* Liberibacter” species. The alignment shows a comparison of type 4 prophage-like sequence Ishi-1-a to genomes of “*Ca.* Liberibacter africanus” ( GCA_001021085.1) and “*Ca.* Liberibacter americanus” ( GCA_000496595.1).

To assess the evolutionary origin of the type 4 prophage-like sequence and its time of insertion into the bacterial genome, we searched for type 4 sequences in “*Ca.* Liberibacter asiaticus”-related species. The uncultivated “*Candidatus* Liberibacter africanus” and “*Candidatus* Liberibacter americanus” species are closely related to “*Ca.* Liberibacter asiaticus” and have been identified as less-prevalent causative agents of HLB (1). According to nucleotide and protein alignments, the prophage-like sequence of type 4 is partially present in both “*Ca*. Liberibacter africanus” and “*Ca.* Liberibacter americanus” (Fig. 4). The presence of the type 4 sequence in these genomes could suggest that the integration of the type 4 prophage-like sequence in the Liberibacter genome occurred 309 million years (myr) ago or earlier (estimated time of lineage divergence for the “*Ca.* Liberibacter asiaticus”/“*Ca.* Liberibacter africanus” and the “*Ca.* Liberibacter americanus” clades [21]). To test this hypothesis, we compared the average nucleotide identity (ANI) and average amino acid identity (AAI) values determined for whole genomes to that of the prophage-like sequences. We observed high similarity in the ANI values determined for “*Ca.* Liberibacter africanus” and the Ishi-1 strain (81.60%) and in the ANI values determined for the corresponding predicted type 4 prophage-like sequences (81.69%). Similarly, the AAI values determined for “*Ca.* Liberibacter americanus” and “*Ca.* Liberibacter africanus,” for “*Ca.* Liberibacter americanus” and Ishi-1, and for “*Ca.* Liberibacter africanus” and Ishi-1 (65.8%, 66.29%, and 76.79%, respectively) match the AAI values determined for the corresponding predicted type 4 prophage-like sequences (64.52%, 61.9%, and 77.83%, respectively). These findings are consistent with the integration of a type 4 prophage-like sequence prior to lineage divergence. The presence of a type 4 sequence in almost all evaluated genomes challenges the common view of “*Ca.* Liberibacter asiaticus” strains lacking prophage or prophage-like sequences.

## DISCUSSION

SC1 and SC2 phages were first discovered in 2011 (7). Since then, the release of a new “*Ca.* Liberibacter asiaticus” genome has usually included analysis of prophage types (e.g., 10, 11, 15), whether of type 1, 2, or 3. These phage types can be used to compare evolutionary dynamics (9, 12) and to determine the origin of strains (9, 14, 17). Nonetheless, prior work has leveraged read-mapping-based approaches to identify new prophage-like sequences, albeit those that resemble type 1 or 2 phage. Indeed, even the discovery of a type 3 prophage (13) was due to an incomplete mapping of the known phages. This bias has led to categorization of multiple strains as lacking prophage (8, 13, 16) and to partial reduction of the importance of prophage sequences in analyses of the pathogenicity of “*Ca.* Liberibacter asiaticus” (8).

Here, we utilized a *de novo*-based search to identify new prophage sequences in “*Ca.* Liberibacter asiaticus” genomes. Our strategy allowed us to confirm the presence of publicly available phage genomes and of new prophages of types 1, 2, and 3, as well as of novel prophage-like sequences that did not resemble previously identified phage types. Subsequent classification analysis identified 12 sequences as part of new type 4 prophage-like sequences that are found in 12 of the 15 “*Ca.* Liberibacter asiaticus” strains. Sequence alignment and gene content analyses support the hypothesis that type 4 represents a new sequence unrelated to the previously identified prophages. Several characteristics suggest that type 4 sequences are remnants of a prophage integrated in the bacterial genome. These characteristics include the disruption of viral-particle-forming genes, the sequence size, the G+C content closely resembling that of the bacterial host, and the failure to find potential att sites. The presence of a type 4 prophage-like sequence in nearly all “*Ca.* Liberibacter asiaticus” genomes suggests that further studies of the relationship between prophage—whether active or remnant—and bacterial pathogenicity are warranted.

In summary, by adopting a *de novo* prediction approach, we have significantly expanded the diversity of prophage-like sequences that have been identified in “*Ca.* Liberibacter asiaticus.” Moving forward, combining reference-based and *de novo* approaches is likely to contribute to understanding of the diversity, function, and evolution of phage on “*Ca.* Liberibacter asiaticus” bacteria. As in other comparative studies of phage, many of the functions of genes in prophage and prophage-like sequences remain unknown. Investigation of the functions of the hypothetical proteins present in prophage-like sequences in bacterial genomes might further understanding of the mechanisms of “*Ca.* Liberibacter asiaticus” virulence and pathogenicity. We hope that the present report of “*Ca.* Liberibacter asiaticus” and associated prophage-like sequences will help provide a new approach to identify both causes of and solutions to Huanglongbing disease.

## MATERIALS AND METHODS

### Genome sequences.

A total of 15 genome sequences from different “*Ca.* Liberibacter asiaticus” strains were retrieved from the National Center for Biotechnology Information (NCBI) Nucleotide Database, under genome identifier (ID) 1750 (Table 1) (https://www.ncbi.nlm.nih.gov/genome/genomes/1750). All “*Ca.* Liberibacter asiaticus” prophages previously reported were also retrieved from NCBI (Table 2).

### Prophage-like sequence identification.

All “*Ca.* Liberibacter asiaticus” genomes were examined for prophage sequences using the phage identification tools Virsorter (18) and PHASTER (19). The genomes were imported into Virsorter (18) in the Discovery Environment (CyVerse) and evaluated against the available Refseq database. As PHASTER works only for sequences larger than 1,500 bp, contigs with a size smaller than the required were discarded. Only prophage-like sequences predicted by both tools were kept for further analysis.

### Phage classification.

Classification of putative prophages began with a comparison to phage genomes available in NCBI. Predicted prophages that matched publicly available phage were not subjected to further classification. In all other cases, SC1, SC2, and P-JXGC-3 phages were used as phages representative of type 1, type 2, and type 3. Two-way nucleotide BLAST analyses were performed with default parameters with the predicted sequence as query or target. Coverage and identity values were divided in high, intermediate, or low categories. Identity (I) was considered high with I values ≥ 95, intermediate with I values < 95 and ≥ 85, and low with I values < 85. Two sets of thresholds were used to evaluate the coverage. When the query sequence was shorter than the target, the coverage (C) was considered high with C values ≥ 90, intermediate with C values < 90 and ≥ 75, and low with C values < 75. When the query sequence was longer than the target, the coverage was considered high with C values ≥ 33, intermediate with C values < 33 and ≥ 25, and low with C values < 25. A sequence was classified as a particular phage type if none of its BLAST values were “low” and if it had the highest fraction of “high” scored values. If more than one phage type fulfilled the aforementioned conditions, the assigned phage type was associated with that with the highest identity value. The putative prophages that were not classified as type 1, 2, or 3 were aligned against each other as part of the process of identifying a potential (new) type 4 group.

### CDS prediction, sequence clusterization, and gene annotation.

For sequence annotation, open reading frames (ORFs) were identified with GeneMarkS-2 (22). Data representing homology between predicted proteins were obtained using get_homologues software (23) with default parameters for the bidirectional best hit. The presence or absence of genes in the pangenome was hierarchically clustered in R to group prophage-like sequences according to their shared CDS. Default parameters for the heatmap.2 function from the gplot package (version 3.0.1.1), including “average” linkage, were used. The protein predictions were annotated with BLAST against the nr database. The Tandem Repeats Finder (24) was used to find potential attachment sites 5,000 bp upstream and 5,000 bp downstream from the predicted prophage sequences. Additionally, average nucleotide identity (ANI) and average amino acid identity (AAI) were calculated using the enveomics collection (25).

### Data availability.

R (v3.3) and Bash were used to generate all figures. Code is available at https://github.com/WeitzGroup/CLas_prophages with zenodo link at https://doi.org/10.5281/zenodo.3405598.

## ADDENDUM IN PROOF

At the proof stage, we learned that Chen et al. (15) had issued an erratum in which they corrected their classification and identified a type 1 (rather than type 2) sequence, which is consistent with our findings.
