# Supplementary material for: Functional and Comparative Genomic Analysis of Integrated Prophage-Like Sequences in “Candidatus Liberibacter asiaticus”
Source: mSphere. 2019 Nov 13;4(6):e00409-19. doi: 10.1128/mSphere.00409-19 (PMC6854039; doi:10.1128/mSphere.00409-19)
Supplement: TABLE S1 [file mSphere.00409-19-st001.docx]

|  | | | **A4-a** | **AHCA1-a (Fragment)** | **FL17-a (Fragment)** | **HHCA1-a** | **JXGC-a** | **TX1712-a** | **YCPsy-a** | **YNJS7C-a** | **gxpsy-a** | **Ishi-1-a** | **psy62-c** | **SGCA5-a** |
| --- | --- | --- | --- | --- | --- | --- | --- | --- | --- | --- | --- | --- | --- | --- |
| **A4-a** | Target | C | 100 | 100 | 99 | 100 | 100 | 96 | 100 | 96 | 100 | 96 | 96 | 100 |
|  |  | I | 100 | 99.8 | 99.7 | 99.2 | 99.8 | 99.7 | 99.8 | 99.8 | 99.8 | 99.8 | 99.7 | 99.8 |
|  | Query | C | 100 | 72 | 76 | 33 | 100 | 87 | 100 | 87 | 100 | 87 | 87 | 22 |
|  |  | I | 100 | 99.8 | 99.7 | 99.2 | 99.8 | 99.7 | 99.8 | 99.8 | 99.8 | 99.8 | 99.7 | 99.8 |
| **AHCA1-a**  **(Fragment)** | Target | C | 72 | 100 | 95 | 100 | 72 | 81 | 72 | 81 | 72 | 81 | 81 | 100 |
|  |  | I | 99.8 | 100 | 99.9 | 99.6 | 99.9 | 99.9 | 99.9 | 99.9 | 99.9 | 99.9 | 99.9 | 99.9 |
|  | Query | C | 100 | 100 | 100 | 45 | 100 | 100 | 100 | 100 | 100 | 100 | 100 | 30 |
|  |  | I | 99.8 | 100 | 99.9 | 99.6 | 99.9 | 99.9 | 99.9 | 99.9 | 99.9 | 99.9 | 99.9 | 99.9 |
| **FL17-a**  **(Fragment)** | Target | C | 76 | 100 | 100 | 100 | 76 | 85 | 76 | 85 | 76 | 85 | 85 | 100 |
|  |  | I | 99.7 | 99.9 | 100 | 99.7 | 100 | 99.9 | 100 | 99.9 | 99.9 | 99.9 | 99.9 | 99.9 |
|  | Query | C | 99 | 95 | 100 | 43 | 99 | 99 | 99 | 99 | 99 | 99 | 99 | 28 |
|  |  | I | 99.7 | 99.9 | 100 | 99.7 | 100 | 99.9 | 100 | 99.9 | 99.9 | 99.9 | 99.9 | 99.9 |
| **HHCA1-a** | Target | C | 33 | 45 | 43 | 100 | 33 | 36 | 33 | 36 | 33 | 36 | 36 | 34 |
|  |  | I | 99.2 | 99.6 | 99.7 | 100 | 99.7 | 99.7 | 99.7 | 99.7 | 99.7 | 99.7 | 99.7 | 99 |
|  | Query | C | 100 | 100 | 100 | 100 | 100 | 100 | 100 | 100 | 100 | 100 | 100 | 22 |
|  |  | I | 99.2 | 99.6 | 99.7 | 100 | 99.7 | 99.7 | 99.7 | 99.7 | 99.7 | 99.7 | 99.7 | 99 |
| **JXGC-a** | Target | C | 100 | 100 | 99 | 100 | 100 | 96 | 100 | 96 | 100 | 96 | 96 | 100 |
|  |  | I | 99.8 | 99.9 | 100 | 99.7 | 100 | 100 | 100 | 100 | 100 | 100 | 100 | 99.9 |
|  | Query | C | 100 | 72 | 76 | 33 | 100 | 87 | 100 | 87 | 100 | 87 | 87 | 22 |
|  |  | I | 99.8 | 99.9 | 100 | 99.7 | 100 | 100 | 100 | 100 | 100 | 100 | 100 | 99.9 |
| **TX1712-a** | Target | C | 87 | 100 | 99 | 100 | 87 | 100 | 87 | 100 | 87 | 100 | 100 | 100 |
|  |  | I | 99.7 | 99.9 | 99.9 | 99.7 | 100 | 100 | 100 | 100 | 100 | 100 | 100 | 99.9 |
|  | Query | C | 96 | 81 | 85 | 36 | 96 | 100 | 96 | 100 | 96 | 100 | 100 | 24 |
|  |  | I | 99.7 | 99.9 | 99.9 | 99.7 | 100 | 100 | 100 | 100 | 100 | 100 | 100 | 99.9 |
| **YCPsy-a** | Target | C | 100 | 100 | 99 | 100 | 100 | 96 | 100 | 96 | 100 | 96 | 96 | 100 |
|  |  | I | 99.8 | 99.9 | 100 | 99.7 | 100 | 100 | 100 | 100 | 100 | 100 | 100 | 99.9 |
|  | Query | C | 100 | 72 | 76 | 33 | 100 | 87 | 100 | 87 | 100 | 87 | 87 | 22 |
|  |  | I | 99.8 | 99.9 | 100 | 99.7 | 100 | 100 | 100 | 100 | 100 | 100 | 100 | 99.9 |
| **YNJS7C-a** | Target | C | 87 | 100 | 99 | 100 | 87 | 100 | 87 | 100 | 87 | 100 | 100 | 100 |
|  |  | I | 99.8 | 99.9 | 99.9 | 99.7 | 100 | 100 | 100 | 100 | 100 | 100 | 100 | 99.9 |
|  | Query | C | 96 | 81 | 85 | 36 | 96 | 100 | 96 | 100 | 96 | 100 | 100 | 24 |
|  |  | I | 99.8 | 99.9 | 99.9 | 99.7 | 100 | 100 | 100 | 100 | 100 | 100 | 100 | 99.9 |
| **gxpsy-a** | Target | C | 100 | 100 | 99 | 100 | 100 | 96 | 100 | 96 | 100 | 96 | 96 | 100 |
|  |  | I | 99.8 | 99.9 | 99.9 | 99.7 | 100 | 100 | 100 | 100 | 100 | 100 | 99.9 | 99.9 |
|  | Query | C | 100 | 72 | 76 | 33 | 100 | 87 | 100 | 87 | 100 | 87 | 87 | 22 |
|  |  | I | 99.8 | 99.9 | 99.9 | 99.7 | 100 | 100 | 100 | 100 | 100 | 100 | 99.9 | 99.9 |
| **Ishi-1-a** | Target | C | 87 | 100 | 99 | 100 | 87 | 100 | 87 | 100 | 87 | 100 | 100 | 100 |
|  |  | I | 99.8 | 99.9 | 99.9 | 99.7 | 100 | 100 | 100 | 100 | 100 | 100 | 100 | 99.9 |
|  | Query | C | 96 | 81 | 85 | 36 | 96 | 100 | 96 | 100 | 96 | 100 | 100 | 24 |
|  |  | I | 99.8 | 99.9 | 99.9 | 99.7 | 100 | 100 | 100 | 100 | 100 | 100 | 100 | 99.9 |
| **psy62-c** | Target | C | 87 | 100 | 99 | 100 | 87 | 100 | 87 | 100 | 87 | 100 | 100 | 100 |
|  |  | I | 99.7 | 99.9 | 99.9 | 99.7 | 100 | 100 | 100 | 100 | 99.9 | 100 | 100 | 99.9 |
|  | Query | C | 96 | 81 | 85 | 36 | 96 | 100 | 96 | 100 | 96 | 100 | 100 | 24 |
|  |  | I | 99.7 | 99.9 | 99.9 | 99.7 | 100 | 100 | 100 | 100 | 99.9 | 100 | 100 | 99.9 |
| **SGCA5-a** | Target | C | 22 | 30 | 28 | 22 | 22 | 24 | 22 | 24 | 22 | 24 | 24 | 100 |
|  |  | I | 99.8 | 99.9 | 99.9 | 99 | 99.9 | 99.9 | 99.9 | 99.9 | 99.9 | 99.9 | 99.9 | 100 |
|  | Query | C | 100 | 100 | 100 | 34 | 100 | 100 | 100 | 100 | 100 | 100 | 100 | 100 |
|  |  | I | 99.8 | 99.9 | 99.9 | 99 | 99.9 | 99.9 | 99.9 | 99.9 | 99.9 | 99.9 | 99.9 | 100 |
