# Supplementary material for: Functional and Comparative Genomic Analysis of Integrated Prophage-Like Sequences in “Candidatus Liberibacter asiaticus”
Source: mSphere. 2019 Nov 13;4(6):e00409-19. doi: 10.1128/mSphere.00409-19 (PMC6854039; doi:10.1128/mSphere.00409-19)
Supplement: TABLE S2 [file mSphere.00409-19-st002.docx]

| **ORF** | **Begin** | **End** | **UniRef ID** | **Function** | **locus_tag** | **Begin** | **End** | **Function** |
| --- | --- | --- | --- | --- | --- | --- | --- | --- |
| **ORF1** | 1 | 312 | WP_015452960.1 | hypothetical protein | CGUJ_05155 | 1127556 | 1127867 | hypothetical protein |
| **ORF2** | 602 | 1738 | WP_015452961.1 | hypothetical protein | CGUJ_05160 | 1128157 | 1129293 | hypothetical protein |
| **ORF3** | 1876 | 2328 | WP_045490404.1 | hypothetical protein | CGUJ_05165 | 1129431 | 1129883 | hypothetical protein |
| **ORF4** | 2332 | 3048 | KIH96222.1 | hypothetical protein | CGUJ_05170 | 1130246 | 1130341 | hypothetical protein |
| **ORF5** | 3303 | 3782 | WP_015452967.1 | hypothetical protein | CGUJ_05175 | 1130858 | 1131337 | hypothetical protein |
| **ORF6** | 3785 | 4807 | ONI58489.1 | hypothetical protein | CGUJ_05180 | 1131340 | 1132302 | hypothetical protein |
|  |  |  |  |  | CGUJ_05185 | 1132338 | 1132589 | hypothetical protein |
| **ORF7** | 4783 | 5034 |  |  |  |  |  |  |
| **ORF8** | 5031 | 5375 |  |  | CGUJ_05190 | 1132586 | 1132930 | hypothetical protein |
| **ORF9** | 5377 | 5964 | WP_015452969.1 | hypothetical protein | CGUJ_05195 | 1132932 | 1133519 | hypothetical protein |
| **ORF10** | 5974 | 6510 | WP_015452970.1 | hypothetical protein | CGUJ_05200 | 1133529 | 1134065 | hypothetical protein |
| **ORF11** | 6511 | 6708 | WP_015824980.1 | hypothetical protein | CGUJ_05205 | 1134066 | 1134239 | hypothetical protein |
| **ORF12** | 6846 | 7334 | WP_076969211.1 | head protein |  | | | |
| **ORF13** | 7331 | 7972 |  |  |  | | | |
| **ORF14** | 8015 | 8605 | WP_015452973.1 | hypothetical protein | CGUJ_05225 | 1135570 | 1136160 | hypothetical protein |
|  |  |  |  |  | CGUJ_05230 | 1136411 | 1136659 | hypothetical protein |
| **ORF15** | 9234 | 9341 | WP_076969212.1 | phage portal protein | CGUJ_05235 | 1136789 | 1136896 | hypothetical protein |
| **ORF16** | 9332 | 9562 |  |  | CGUJ_05240 | 1136887 | 1137117 | hypothetical protein |
| **ORF17** | 9572 | 10624 |  |  | CGUJ_05245 | 1137127 | 1138179 | hypothetical protein |
| **ORF18** | 10874 | 11629 | WP_083965928.1 | DNA packaging protein |  | | | |
| **ORF19** | 11616 | 11948 |  |  |  | | | |
| **ORF20** | 12019 | 12387 | WP_015452976.1 | hypothetical protein | CGUJ_05265 | 1139574 | 1139942 | hypothetical protein |
| **ORF22** | 13941 | 14192 | WP_015452977.1 | XRE family transcriptional regulator | CGUJ_05270 | 1141496 | 1141747 | transcriptional regulator |
| **ORF23** | 15159 | 15959 | WP_076969216.1 | DNA helicase | CGUJ_05280 | 1142714 | 1143514 | replicative DNA helicase |
| **ORF24** | 16355 | 16456 |  |  | CGUJ_05285 | 1143910 | 1144011 | hypothetical protein |
| **ORF25** | 16450 | 16566 | WP_076969217.1 | DUF1376 domain-containing protein | CGUJ_05290 | 1144005 | 1144121 | hypothetical protein |
|  |  |  |  |  | CGUJ_05295 | 1144105 | 1144572 | hypothetical protein |
| **ORF26** | 16559 | 17017 | WP_076778991.1 | DUF1376 domain-containing protein |  |  |  |  |
