# Supplementary material for: Functional and Comparative Genomic Analysis of Integrated Prophage-Like Sequences in “Candidatus Liberibacter asiaticus”
Source: mSphere. 2019 Nov 13;4(6):e00409-19. doi: 10.1128/mSphere.00409-19 (PMC6854039; doi:10.1128/mSphere.00409-19)
Supplement: TABLE S3 [file mSphere.00409-19-st003.docx]

| **ORF** | **Begin** | **End** | **UniRef ID** | **Function** | **locus_tag** | **Begin** | **End** | **Function** |
| --- | --- | --- | --- | --- | --- | --- | --- | --- |
| **ORF1** | 1 | 480 | WP_015452967.1 | hypothetical protein | DIC79_05165 | 1135796 | 1136275 | hypothetical protein |
| **ORF2** | 483 | 1445 | WP_015824978.1 | hypothetical protein | DIC79_05170 | 1136278 | 1137240 | hypothetical protein |
| **ORF3** | 1481 | 1732 | WP_015824979.1 | hypothetical protein | DIC79_05175 | 1137276 | 1137527 | hypothetical protein |
| **ORF4** | 1729 | 2073 | WP_015452968.1 | hypothetical protein | DIC79_05180 | 1137524 | 1137868 | hypothetical protein |
| **ORF5** | 2075 | 2662 | WP_015452969.1 | hypothetical protein | DIC79_05185 | 1137870 | 1138457 | hypothetical protein |
| **ORF6** | 2672 | 3208 | WP_015452970.1 | hypothetical protein | DIC79_05190 | 1138467 | 1139003 | hypothetical protein |
| **ORF7** | 3209 | 3382 | WP_015824980.1 | hypothetical protein | DIC79_05195 | 1139004 | 1139201 | hypothetical protein |
| **ORF9** | 3544 | 4032 | WP_076969211.1 | head protein | DIC79_05200 | 1139339 | 1140333 | head protein |
| **ORF10** | 4029 | 4538 |  |  |  |  |  |  |
| **ORF11** | 4713 | 5303 | WP_015452973.1 | hypothetical protein | DIC79_05205 | 1140508 | 1141098 | hypothetical protein |
| **ORF13** | 5554 | 5802 | WP_015452974.1 | hypothetical protein | DIC79_05210 | 1141349 | 1141597 | hypothetical protein |
| **ORF14** | 5932 | 6039 | WP_076969212.1 | phage portal protein |  | | | |
| **ORF15** | 6030 | 6260 | WP_076969212.1 | phage portal protein | DIC79_05215 | 1141825 | 1142055 | hypothetical protein |
| **ORF16** | 6270 | 7322 | WP_076969212.1 | phage portal protein | DIC79_05220 | 1142065 | 1143093 | hypothetical protein |
| **ORF17** | 7399 | 7587 | WP_083965928.1 | DNA packaging protein | DIC79_05225 | 1143194 | 1144561 | DNA packaging protein |
| **ORF18** | 7572 | 8327 |  |  |  |  |  |  |
| **ORF19** | 8314 | 8463 |  |  |  |  |  |  |
| **ORF20** | 8512 | 8766 |  |  |  |  |  |  |
|  |  |  |  |  | DIC79_05230 | 1144512 | 1144880 | hypothetical protein |
| **ORF21** | 8717 | 9085 | WP_015452976.1 | hypothetical protein | DIC79_05225 | 1143194 | 1144561 | DNA packaging protein |
|  |  |  |  |  | DIC79_05230 | 1144512 | 1144880 | hypothetical protein |
| **ORF22** | 10638 | 10889 | WP_015452977.1 | XRE family TR | DIC79_05235 | 1146433 | 1146684 | XRE family TR |
| **ORF23** | 11723 | 11866 | WP_047263802.1 | DNA helicase | DIC79_05240 | 1147500 | 1148948 | DNA helicase |
| **ORF24** | 11856 | 12656 | WP_015824985.1 | DNA helicase |  |  |  |  |
| **ORF25** | 12966 | 13112 | OMH86553.1 | head protein partial |  | | | |
| **ORF26** | 13147 | 13263 | WP_047263803.1 | DUF1376 domain protein | DIC79_05245 | 1149012 | 1149509 | DUF1376 domain protein |
| **ORF27** | 13256 | 13714 | WP_076778991.1 | DUF1376 domain protein |  |  |  |  |
| **ORF28** | 14155 | 14247 | AGH16429.1 | ribonucleotide-diphosphate reductase subunit beta |  | | | |
| **ORF29** | 14546 | 14773 | WP_041862253.1 | hypothetical protein |  | | | |
| **ORF30** | 14798 | 14995 | WP_015452983.1 | hypothetical protein | DIC79_05250 | 1150593 | 1150790 | hypothetical protein |
| **ORF31** | 15052 | 15192 | ACT57628.1 | hypothetical protein |  | | | |
| **ORF32** | 15592 | 16056 | WP_015452985.1 | hypothetical protein | DIC79_05255 | 1151387 | 1151851 | hypothetical protein |
|  | | | | | DIC79_05260 | 1151966 | 1152228 | hypothetical protein |
| **ORF33** | 17434 | 19329 | WP_015452987.1 | peptidyl-prolyl cis-trans isomerase | DIC79_05265 | 1153229 | 1155124 | peptidylprolyl isomerase |
|  | | | | | DIC79_05270 | 1155612 | 1155815 | hypothetical protein |
| **ORF34** | 20953 | 21690 | WP_015452989.1 | 16S rRNA (uracil(1498)-N(3))-methyltransferase | DIC79_05275 | 1156748 | 1157485 | 16S rRNA (uracil(1498)-N(3))-methyltransferase |
| **ORF35** | 21761 | 23134 | WP_015452990.1 | glutamate--cysteine ligase | DIC79_05280 | 1157556 | 1158929 | glutamate--cysteine ligase |
| **ORF36** | 23168 | 23668 | WP_015452991.1 | N-acetyltransferase | DIC79_05285 | 1158963 | 1159403 | N-acetyltransferase |
| **ORF37** | 23944 | 24588 | WP_015452992.1 | hypothetical protein | DIC79_05290 | 1159739 | 1160383 | hypothetical protein |
| **ORF38** | 25614 | 25793 | ACT57637.1 | hypothetical protein |  | | | |
| **ORF39** | 25806 | 26411 | WP_015452994.1 | pyridoxamine 5'-phosphate oxidase | DIC79_05295 | 1161601 | 1162206 | pyridoxamine 5'-phosphate oxidase |
| **ORF40** | 26554 | 27408 | WP_015452995.1 | enoyl-ACP reductase | DIC79_05300 | 1162349 | 1163203 | enoyl-[acyl-carrier-protein] reductase FabI |
| **ORF41** | 27704 | 28756 | WP_015452996.1 | tRNA dihydrouridine(20/20a) synthase DusA | DIC79_05305 | 1163499 | 1164551 | tRNA dihydrouridine(20/20a) synthase DusA |
| **ORF42** | 29086 | 30471 | WP_015452997.1 | PTS ascorbate transporter subunit IIC | DIC79_05310 | 1164881 | 1166266 | PTS ascorbate transporter subunit IIC |
| **ORF43** | 30511 | 30633 | WP_031935092.1 | hypothetical protein |  | | | |
| **ORF44** | 30713 | 32164 | WP_015452998.1 | deoxyribodipyrimidine photo-lyase | DIC79_05315 | 1166508 | 1167959 | deoxyribodipyrimidine photo-lyase |
| **ORF45** | 32429 | 33691 | WP_015452999.1 | dicarboxylate/amino acid:cation symporter | DIC79_05320 | 1168224 | 1169486 | dicarboxylate/amino acid:cation symporter |
| **ORF46** | 33785 | 35980 | WP_015453000.1 | NAD-dependent DNA ligase LigA | DIC79_05325 | 1169580 | 1171775 | NAD-dependent DNA ligase LigA |
| **ORF47** | 36098 | 37765 | WP_015453001.1 | DNA repair protein RecN | DIC79_05330 | 1171893 | 1173560 | DNA repair protein RecN |
| **ORF48** | 37827 | 38642 | WP_015453002.1 | outer membrane protein assembly factor BamD | DIC79_05335 | 1173622 | 1174437 | outer membrane protein assembly factor BamD |
| **ORF49** | 38769 | 39659 | WP_015824991.1 | UDP-3-O-acyl-N-acetylglucosamine deacetylase | DIC79_05340 | 1174564 | 1175454 | UDP-3-O-acyl-N-acetylglucosamine deacetylase |
| **ORF50** | 39749 | 41257 | WP_015453004.1 | cell division protein FtsZ | DIC79_05345 | 1175544 | 1177052 | cell division protein FtsZ |
| **ORF51** | 41360 | 42682 | WP_015453005.1 | cell division protein FtsA | DIC79_05350 | 1177155 | 1178477 | cell division protein FtsA |
| **ORF52** | 42682 | 43596 | WP_015453006.1 | cell division protein | DIC79_05355 | 1178477 | 1179391 | cell division protein |
|  |  |  |  |  | DIC79_05360 | 1179379 | 1180296 | D-alanine--D-alanine ligase |
| **ORF53** | 43584 | 44519 | WP_015453007.1 | D-alanine--D-alanine ligase | DIC79_05355 | 1178477 | 1179391 | cell division protein |
|  |  |  |  |  | DIC79_05360 | 1179379 | 1180296 | D-alanine--D-alanine ligase |
| **ORF54** | 45122 | 45337 | WP_015453008.1 | hypothetical protein | DIC79_05365 | 1180917 | 1181132 | hypothetical protein |
| **ORF55** | 45453 | 45710 | WP_015453009.1 | hypothetical protein | DIC79_05370 | 1181248 | 1181505 | hypothetical protein |
| **ORF57** | 46336 | 46620 | WP_015453010.1 | DUF4145 domain protein | DIC79_05375 | 1182131 | 1182415 | DUF4145 domain protein |
| **ORF58** | 46765 | 46977 | WP_015453011.1 | hypothetical protein | DIC79_05380 | 1182560 | 1182844 | hypothetical protein |
| **ORF59** | 47803 | 48039 | WP_015453012.1 | hypothetical protein | DIC79_05385 | 1183598 | 1183834 | hypothetical protein |
| **ORF61** | 49539 | 50642 | WP_015453015.1 | terminase | DIC79_05390 | 1185334 | 1186437 | terminase |
| **ORF62** | 51030 | 51587 | WP_015453016.1 | hypothetical protein | DIC79_05395 | 1186825 | 1187382 | hypothetical protein |
| **ORF63** | 53255 | 54016 | WP_015453017.1 | hypothetical protein | DIC79_05400 | 1189050 | 1189808 | hypothetical protein |
|  |  |  |  |  | DIC79_05405 | 1189768 | 1190469 | hypothetical protein |
| **ORF64** | 53973 | 54674 | WP_015453018.1 | hypothetical protein partial | DIC79_05400 | 1189050 | 1189808 | hypothetical protein |
|  |  |  |  |  | DIC79_05405 | 1189768 | 1190469 | hypothetical protein |
| **ORF65** | 54700 | 55197 | WP_015453019.1 | DUF2800 domain protein | DIC79_05410 | 1190495 | 1190992 | DUF2800 domain protein |
| **ORF66** | 55202 | 55798 | WP_015453020.1 | DUF2815 domain protein | DIC79_05415 | 1190997 | 1191593 | DUF2815 domain protein |
| **ORF67** | 55801 | 57828 | WP_015824993.1 | DNA polymerase | DIC79_05420 | 1191596 | 1193623 | DNA polymerase |
| **ORF68** | 57825 | 58121 | WP_015824994.1 | nuclease | DIC79_05425 | 1193620 | 1193916 | nuclease |
| **ORF69** | 58112 | 59494 | WP_040055333.1 | ATP-dependent helicase | DIC79_05430 | 1193907 | 1195289 | ATP-dependent helicase |
| **ORF70** | 59487 | 59846 | WP_012778351.1 | DNA ligase | DIC79_05435 | 1195282 | 1195641 | DNA ligase |
| **ORF71** | 59848 | 60420 | WP_015453025.1 | guanylate kinase | DIC79_05440 | 1195643 | 1196215 | guanylate kinase |
| **ORF72** | 60492 | 60800 | YP_007011137.1 | hypothetical protein | DIC79_05445 | 1196287 | 1196596 | hypothetical protein |
